# Dynamic Response of a Rigid Pavement Plate Based on an Inertial Soil

**DOI:** 10.1155/2016/4975345

**Published:** 2016-01-10

**Authors:** Mohamed Gibigaye, Crespin Prudence Yabi, I. Ezéchiel Alloba

**Affiliations:** University of Abomey-Calavi, 01 BP 2009 Cotonou, Benin

## Abstract

This work presents the dynamic response of a pavement plate resting on a soil whose inertia is taken into account in the design of pavements by rational methods. Thus, the pavement is modeled as a thin plate with finite dimensions, supported longitudinally by dowels and laterally by tie bars. The subgrade is modeled via Pasternak-Vlasov type (three-parameter type) foundation models and the moving traffic load is expressed as a concentrated dynamic load of harmonically varying magnitude, moving straight along the plate with a constant acceleration. The governing equation of the problem is solved using the modified Bolotin method for determining the natural frequencies and the wavenumbers of the system. The orthogonal properties of eigenfunctions are used to find the general solution of the problem. Considering the load over the center of the plate, the results showed that the deflections of the plate are maximum about the middle of the plate but are not null at its edges. It is therefore observed that the deflection decreased 18.33 percent when the inertia of the soil is taken into account. This result shows the possible economic gain when taking into account the inertia of soil in pavement dynamic design.

## 1. Introduction

Pavements are an essential feature of the urban communication system and provide an efficient means of transportation of goods and services. Depending on its rigidity compared to the subsoil, pavements are classified as flexible, rigid, and semiflexible [1]. To design these classes of pavements, the rational methods models are often used. The backbone of a method of roadway design is the mechanical model used to define the structure [2].

In the case of rigid pavements, the most used models are the multilayer elastic model of Burmister [3] and the Westergaard model, assuming pavement as a plate resting on the Winkler soil type [2, 4–6]. The differences between the pavement and the soil rigidities were conducted by Ullidtz [7], to deduce that Burmister model is generally not considered as an appropriate tool for the analysis of a rigid pavement response. Then, the large used design model of existing rigid pavement is Westergaard's, using the Winkler soil type. Although Winkler model leads to relatively simplified results, it has serious limitations [1]. Firstly, there is the deflection discontinuity between the charged and the uncharged part of pavement plate. Secondly, both models consider loads usually as a static one applied on a plate [2–4, 8]. According to Sun and Greenberg (2000) cited by St-Laurent [9], traffic loads on the pavement induce inertial effects that must be supported by foresaid pavement. Thus, the static load model does not reflect accurately the actual conditions of load on the pavement [10]. During the last decade, many researchers have examined the problem assuming the loads as a dynamic one [1, 8, 10]. In the studies mentioned above, the soil used in the structure modeling are Winkler soil type. But, according to the design guide of United States National Cooperative Highway Research Program (NCHRP) [11], the two-parameter Pasternak model is designated in 1998 as the best pavement option to model the foundation of pavements. From that, many researchers base theirs works on using this type of soil [12, 13]. Alisjahbana and Wangsadinata [14] looked at the dynamic analysis of a rigid pavement under mobile load resting on Pasternak soil type. Based on this model, the determination of soil parameters is only based on the elasticity modulus and Poisson's ratio. On the other hand, the Pasternak-Vlasov model that takes into account the logarithmic decrement of soil was used by Rahman and Anam [1] using the finite element method. The study of Rahman also showed that the Pasternak-Vlasov model is more economical than that of Winkler and cannot arbitrarily set the values of the intrinsic characteristics of the soil.

In most models used previously, the dynamic effect is taken into account only by the inertia of the plate [11, 15]. Concerning the soil, inertia is neglected in dynamic modeling of pavement structure. However, Civalek [16] took into account the inertia of the soil but had defined as constant independents values the intrinsic parameters of soil. He concluded that the effect of foundation inertia on the central deflection of the finite plate is not considerable. But according to the results of Pan and Atluri's work [17], in engineering practice, this is still not always the case, and this factor may have significant effects on the dynamic response of the plate modeling the pavement. For these reasons, several studies have tried to modify the Pasternak-Vlasov soil introducing the inertia of the soil to a depth of soil susceptible to dynamic forces applied to the structure. Gibigaye in his work used an inertia soil model foundation for the study of the behavior of shells modeling underground shells [18]. He concluded that it is important to take into account the inertia of the foundation soil on the dynamic response of civil engineering structures. Besides, Dimitrovová [19] noted that the classical formula which predicts a critical velocity of load significantly is overestimated compared to the one experienced in reality. She indicated that this formula should be revised by introducing two important notions: the effective finite depth of the foundation that is dynamically activated and the inertial effect of this activated foundation layer.

This work investigates dynamic response of a rigid pavement resting on an inertial soil. For that, the rigid pavement is modeled as a thin plate with dowels and tie bars in its edges.

In order to take into account its inertia, the soil is modeled as a three-parameter type (*k*_*o*_, *C*_*o*_, and *m*_*o*_, resp., integral characteristics in compression and in shearing and reduced mass of the foundation soil). The boundary conditions of these plates are modeled by two linear relationships between strains and stresses at the plate edges. The homogeneous solution of the problem is achieved by the method of separation of variables, so that the superposition gives a solution satisfying the boundary conditions. Since the deformation is expressed as eigenfunctions products, the solution of the dynamic problem is obtained based on the orthogonality properties of eigenfunctions. The general solution of the problem is obtained from the specific properties of the Dirac delta function. This paper provides an overview of the dynamic analysis of rigid pavements response as described above.

## 2. Materials and Methods

### 2.1. Governing Equation

In this research work, an isotropic homogeneous elastic rectangular plate resting on an elastic three-parameter soil is considered to model a pavement. The adjacent plates are supposed to be joined by dowels and tie bars. Based on the work of Asik [20] and Gibigaye work in the cylindrical axis system [18], the soil response according to the deflection $\overset{-}{w}(x,y,z,t)$ at a given point inside the soil layer is equivalent to

(1) qsx,y,z,t=kow−x,y,z,t−co∇2w−x,y,z,t+mo∂2w−x,y,z,t∂t2,

where *x*, *y*, *z*, and *t* are space-time coordinates of the soil studying point; $\overset{-}{w}(x,y,z,t)$ is the deflection inside the soil layer defined as $\overset{-}{w}\left(x,y,z,t\right)=\overset{-}{w}\left(x,y,0,t\right)\cdot \phi \left(z\right)$; and *ϕ*(*z*) = sinh⁡[*γ*(1 − *z*/*H*_*s*_)]/sinh⁡*γ* is a vertical decay function of soil that must verify *ϕ*(0) = 1 and *ϕ*(*H*_*s*_) = 0; *k*_*o*_, *c*_*o*_, and *m*_*o*_ are, respectively, integral characteristics in compression and in shearing and linear reduced mass of the foundation soil, supposed to be homogeneous and monolayer. They are expressed as follows [21]:

(2) koEo1−υs2∫0Hsϕ′z2dz=Eo2Hs1−υs2γγ+sinh⁡γcosh⁡γsinh⁡γ2,coEo21+υs∫0Hsϕz2dz=EoHs21+υsγsinh⁡γcosh⁡γ−γγsinh⁡γ2,mom∫0Hsϕz2dz=mHssinh⁡γcosh⁡γ−γ2γsinh⁡γ2,

where *H*_*s*_ is the effective finite depth of the foundation that is dynamically activated; *m* is the density of the subgrade; *γ* is a constant, named logarithmic decrement of the soil, which determinates the rate of decrease of the deflections depending on the depth; *E*_*o*_ is Young's Modulus; *υ* is Poisson's ratio.

According to the classic theory of thin plates and if taking into account the reduced mass of soil, the transverse deflection of the Kirchhoff plate satisfies the following partial differential equation:

(3) D∇4wx,y,t+kowx,y,t−co∇2wx,y,t+γh∂wx,y,t∂t+ρh+mo∂2wx,y,t∂t2=px,y,t.

$w(x,y,t)=\overset{-}{w}(x,y,0,t)$ is the deflection of Kirchhoff plate which is equal to the deflection of the plate/soil interface.

*p*(*x*, *y*, *t*) = *p*_*o*_[1 + (1/2)cos⁡(*ω* · *t*)]*δ*[*x* − *x*(*t*)]*δ*[*y* − *y*(*t*)] is the load transmitted to the pavement [14]. Here *x*(*t*) = *v*_*o*_ *t* + (1/2)acc(*t* ^2^); *y*(*t*) = (1/2)*b* are the geometrical position of load at the time *t*; *p*_*o*_ is the magnitude of the moving wheel load; acc is the acceleration of the load; *ω* is the angular frequency of the applied load; *δ* is the Dirac function; *a*, *b*, and *h* are the dimensions of the finite plate and *D* is the flexural stiffness of the plate.

**Figure 1:**
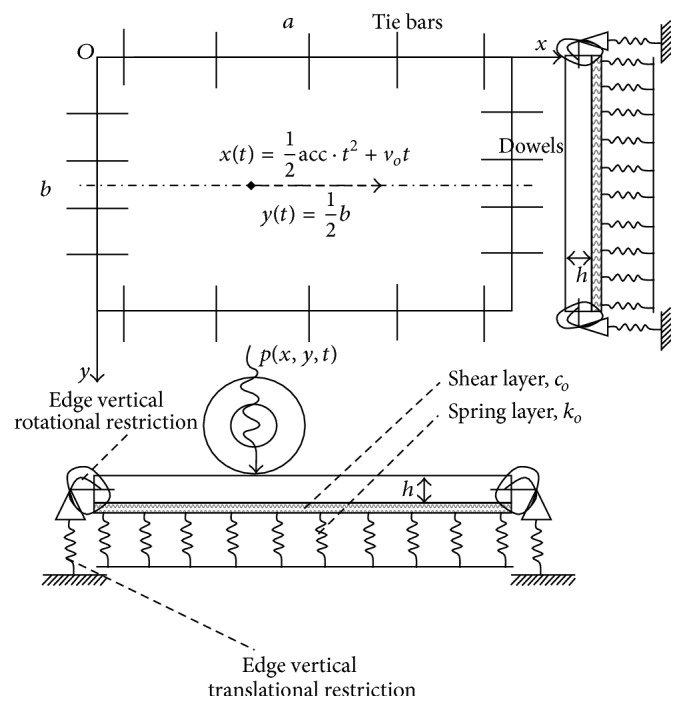
Modeling of doweled rigid pavement under moving load [14].

The boundary conditions (Figure 1) are modeled as follows:

(i) The restriction of the elastic vertical translation is characterized by the four equations [14]:

(4a) Vx=0−D∂3w0,y,t∂x3+2−υ∂3w0,y,t∂x∂y2=ksx1w0,y,t,

(4b) Vx=a−D∂3wa,y,t∂x3+2−υ∂3wa,y,t∂x∂y2=ksx2wa,y,t,

(4c) Vy=0−D∂3wx,0,t∂y3+2−υ∂3wx,0,t∂y∂x2=ksy1wx,0,t,

(4d) Vy=b−D∂3wx,b,t∂y3+2−υ∂3wx,b,t∂y∂x2=ksy2wx,b,t,

where *ks*_*x*1_, *ks*_*x*2_, *ks*_*y*1_, and *ks*_*y*2_ are the elastic vertical translation stiffness and *V*_*x*_, *V*_*y*_ are the vertical shear forces of the plate.

(ii) The restriction of the elastic rotation is characterized by the following four equations [14]:

(5a) Mx=0−D∂2w0,y,t∂x2+υ∂2w0,y,t∂y2=krx1∂∇2w0,y,t∂x,

(5b) Mx=a−D∂2wa,y,t∂x2+υ∂2wa,y,t∂y2=krx2∂∇2wa,y,t∂x,

(5c) My=0−D∂2wx,0,t∂y2+υ∂2wx,0,t∂x2=kry1∂∇2wx,0,t∂y,

(5d) My=b−D∂2wx,b,t∂y2+υ∂2wx,b,t∂x2=kry2∂∇2wx,b,t∂y,

where *kr*_*x*1_, *kr*_*x*2_, *kr*_*y*1_, and *kr*_*y*2_ are the elastic rotational stiffness and *M*_*x*_, *M*_*y*_ are the bending moments of the finite plate.

The initial conditions (*t* = 0 s) are

(6) $$\begin{array}{c} \frac{\partial w\left(x,y,0\right)}{\partial t}=w\left(\;x,y,0\;\right)=0. \end{array}$$

### 2.2. Resolution of the Problem

#### 2.2.1. Determination of the Eigenfrequencies

In order to solve governing equation (3) of the problem, it is assumed that the principal elastic axes of the plate are parallel to its edges. The free vibrations solution of the problem is set as [14, 20]

(7) $$\begin{array}{c} w\left(\;x,y,t\;\right)=\overset{\infty }{\underset{m=1\;}{\sum }}\overset{\infty }{\underset{\;n=1}{\sum }}W_{mn}\left(\;x,y\;\right)\sin\left(\;\omega_{mn}t\;\right). \end{array}$$

*ω*_*mn*_ is the circular frequency of plate and *W*_*mn*_ is the function of position coordinates determined for the mode numbers *m* and *n* in *x*- and *y*-directions. This form satisfies the initial conditions and the undamped free vibrations equation. Therefore natural modes satisfy the equation below [22]:

(8) D∇4wx,y,t+kowx,y,t−co∇2wx,y,t+ρh+mo∂2wx,y,t∂t2=0.

Equation (7) in (8) gives

(9) D∇4Wmn−ρh+moωmn2Wmn+koWmn−co∇2Wmn=0.

This equation is independent of time as the function *W*_*mn*_. Such a problem has solutions which may be in Navier's form [23, 24]:

(10) $$\begin{array}{c} W_{mn}\left(\;x,y\;\right)=A_{mn}\sin\left(\;\frac{p\pi }{a}x\;\right)\sin\left(\;\frac{q\pi }{b}y\;\right). \end{array}$$

Here, *p* and *q* are mode numbers of the plate. They are real numbers because of the boundary conditions of the problem [14, 22, 25] and *m*, *n* are their respective roundness to the nearest integer number. The eigenfrequencies solutions of (9) are for the first auxiliary problem, those which satisfy boundary conditions (5a), (5b), (5c), and (5d):

(11) ωmn2=Dπ4ρh+mopa4+2pqab2+qb4+koρh+mo+coρh+mopπa2+qπb2.

#### 2.2.2. Determination of Eigenmodes of the Plate

To obtain the mode numbers *p* and *q* and the eigenfunctions, modified Bolotin method is used. This consists of the resolution of the two auxiliary Levy's problems. That can permit determining the eigenmode of the plate.

*(i) First Auxiliary Levy Problem*. The solution of (9) for the first auxiliary problem that satisfies the boundary conditions of (4a); (4b); (5a); and (5b) can be expressed as

(12) $$\begin{array}{c} W_{mn}\left(\;x,y\;\right)=X_{mn}\left(\;x\;\right)\sin\left(\;\frac{q\pi }{b}y\;\right), \end{array}$$

where *X*_*mn*_(*x*) is the eigenmode of the plate in the *x*-direction.

Substituting (12) into (9), we obtain an ordinary differential equation for *X*_*mn*_(*y*):

(13) d4Xmnxdx4−2qπb2+coDd2Xmnxdx2−pπa2pπa2+2qπb2+coDXmnx=0.

The solutions of the characteristic equation of (13) are

(14) λ1,2=±πab2q2a2+p2b2+coa2b2Dπ2,λ3,4=±pπai.

For $\beta =\sqrt{2q^{2}a^{2}+p^{2}b^{2}+(c_{o}a^{2}b^{2}/D\pi^{2})}$, *X*(*x*) becomes

(15) Xmnx=A1cosh⁡βπabx+A2sinh⁡βπabx+A3cos⁡pπax+A4sin⁡pπax.

Equation (15) gives the general form of the eigenmode of the plate in the *x*-direction.

Boundary conditions along *x*-axis permit determining the *A*_*i*_ coefficients:

(16) a11A1+a12A2+a13A3+a14A4=0,a21A1+a22A2+a23A3+a24A4=0,a31A1+a32A2+a33A3+a34A4=0,a41A1+a42A2+a43A3+a44A4=0,

where *a*_*ij*_ coefficients are given by

(17) a11=ksx1,a12=Dβπa3−Dβπabqπa2,a13=ksx1,a14=−Dpπa3+2−υpπaqπa2;a21=a12sinh⁡βπb+ksx1sinh⁡βπb,a22=a12cosh⁡βπb+ksx1sinh⁡βπb,a23=−a14sin⁡pπ+ksx1cos⁡pπ,a24=−a14cos⁡pπ+ksx1sin⁡pπ,a31=Dβπab2−υDqπb2;a32=krx1βπab,a33=−Dpπa2+υDqπb2,a34=krx1pπa;a41=a31cosh⁡βπb+krx1βπabsinh⁡βπb,a42=a31sinh⁡βπb+krx1βπabcosh⁡βπb,a43=a33cos⁡pπ−a34sin⁡pπ,a44=a33sin⁡pπ−a34cos⁡pπ.

In order to obtain no trivial solution, it is necessary to propose that the determinant of (16) is zero, so

(18) $$\begin{array}{c} \text{Det}A=0⟹\left|\begin{array}{cccc} a_{11} & a_{12} & a_{13} & a_{14}\\ a_{21} & a_{22} & a_{23} & a_{24}\\ a_{31} & a_{32} & a_{33} & a_{34}\\ a_{41} & a_{42} & a_{43} & a_{44} \end{array}\right|=0. \end{array}$$

*(ii) Second Auxiliary Levy Problem*. The solution of (9) for the second auxiliary problem that satisfies the boundary conditions of (4c); (4d); (5c); and (5d) can be expressed as

(19) $$\begin{array}{c} W_{mn}\left(\;x,y\;\right)=Y_{mn}\left(\;y\;\right)\sin\left(\;\frac{p\pi }{a}x\;\right), \end{array}$$

where *Y*_*mn*_(*y*) is the eigenmode of the plate in the *y*-direction of the plate.

Substituting (19) into (9) conducts to an ordinary differential equation for *Y*_*mn*_(*y*):

(20) d4Ymnydy4−2pπa2+coDd2Ymnydy2−qπb2qπb2+2pπa2+coDYmny=0.

The solutions of the characteristic equation of (20) are

(21) Ymny=B1cosh⁡θπaby+B2sinh⁡θπaby+B3cos⁡qπby+B4sin⁡qπby,

where $\theta =\sqrt{2p^{2}b^{2}+q^{2}a^{2}+(c_{o}a^{2}b^{2}/D\pi^{2})}$.

Equation (21) gives the general form of the eigenmode of the plate in the *y*-direction.

Boundary conditions along *y*-axis permit determining the *B*_*i*_ coefficients:

(22) b11B1+b12B2+b13B3+b14B4=0,b21B1+b22B2+b23B3+b24B4=0,b31B1+b32B2+b33B3+b34B4=0,b41B1+b42B2+b43B3+b44B4=0.

Coefficients *b*_*ij*_ were determined analogously to *a*_*ij*_.

In order to obtain no trivial solution, it is necessary to propose that the determinant of (22) is zero, so

(23) $$\begin{array}{c} \text{Det}B=0⟹\left|\begin{array}{cccc} b_{11} & b_{12} & b_{13} & b_{14}\\ b_{21} & b_{22} & b_{23} & b_{24}\\ b_{31} & b_{32} & b_{33} & b_{34}\\ b_{41} & b_{42} & b_{43} & b_{44} \end{array}\right|=0. \end{array}$$

#### 2.2.3. Determination of Mode Numbers

To obtain the couples {*p*, *q*} that permit having no trivial solutions, the transcendental equation system formed by (18) and (23) is solved. The solution cannot be determined analytically [14] so we used the Wolfram Mathematica software version 8.0.1 to get the numerical solutions. The triangulation of the systems of two equations permits determining the *A*_*i*_ and *B*_*i*_ coefficients after normalizing *A*_1_ and *B*_1_.

The natural mode of the plate is therefore given by

(24) $$\begin{array}{c} W\left(\;x,y\;\right)=\overset{\infty }{\underset{m=1\;}{\sum }}\overset{\infty }{\underset{\;n=1}{\sum }}X_{mn}\left(\;x\;\right)Y_{mn}\left(\;y\;\right). \end{array}$$

#### 2.2.4. Determination of the Time Function *T*_*mn*_

Suppose the solution of governing equation (3) is in the form like

(25) $$\begin{array}{c} W\left(\;x,y,t\;\right)=\overset{\infty }{\underset{m=1\;}{\sum }}\overset{\infty }{\underset{\;n=1}{\sum }}W_{mn}\left(\;x,y\;\right)T_{mn}\left(\;t\;\right), \end{array}$$

where *W*_*mn*_ is a function of the spatial coordinates named modal function or natural mode of the plate and *T*_*mn*_ a function of time.

Thus, for *W*_*mn*_ satisfying (9), the *T*_*mn*_(*t*) function verifies the following equation [22, 24]:

(26) T¨mnt+2αωmnT˙mnt+ωmn2Tmnt=∫0a∫0bWx,ypx,y,tdx dyρh+mo∫0a∫0bWx,y2dx dy

with 2*αω*_*mn*_ = *γh*/(*ρh* + *m*_*o*_) and *α* being the damping ratio of system.

The corresponding homogeneous solutions of (26) can be written:

(27) T0mnt=e−αωmntamncos⁡ωmn1−α2t+bmnsin⁡ωmn1−α2t.

According to the initial conditions defined in (6), *T*_0*mn*_(*t*) = *a*_*mn*_ = *b*_*mn*_ = 0. A particular and the general solution of (26) are both given by [22]

(28) Tmnt=poYmn1/2bρh+moQmnωmn1−α2·∫0t1+12cos⁡ωτXmn12acc·τ2+vo·τ·e−α·ωmnt−τsin⁡ωmn1−α2t−τdτ,

where *Q*_*mn*_ is the normalizing function defined by

(29) $$\begin{array}{c} Q_{mn}=\overset{a}{\underset{0}{\int }}\overset{b}{\underset{0}{\int }}{\left[\;X_{mn}\left(\;x\;\right)\;\right]}^{2}{\left[\;Y_{mn}\left(\;y\;\right)\;\right]}^{2}dx\;dy. \end{array}$$

Finally, the deflection solution of governing equation (3) is in the form of

(30) $$\begin{array}{c} W\left(\;x,y,t\;\right)=\overset{\infty }{\underset{m=1\;}{\sum }}\overset{\infty }{\underset{\;n=1}{\sum }}X_{mn}\left(\;x\;\right)Y_{mn}\left(\;y\;\right)T_{mn}\left(\;t\;\right). \end{array}$$

## 3. Numerical Applications, Results, and Discussion

Using the procedure described above, a rigid roadway pavement subjected to a dynamic traffic load is analyzed. In this work, a finite rectangular plate doweled along its edges is considered as shown in Figure 1. The structural properties of the plate include the size of 5 m × 3.5 m, the thickness of 0.25 m, and physical characteristics of the plate like the density of *ρ* = 2500 kg·m^−3^, Poisson's ratio *υ* = 0.25, and the longitudinal elastic modulus *Ep* = 24.10^9^ Pa. The density of the subgrade is taken equal to *m* = 1800 kg·m^−3^, with Poisson's ratio *υ*_*s*_ = 0.35 and a longitudinal elastic modulus *E* = 50.10^6^ Pa. Finally the moving load magnitude is supposed to be *P*_*o*_ = 80.10^3^ N and circular frequency *ω* = 100 rad·s^−1^, acceleration acc = 2 m·s^−2^, and a speed of *v*_*o*_ = 25 m·s^−1^ [14]. These parameters are typical material and structural properties of highway and airport pavements according to French Central Laboratory of Bridges and Pavements [3]. It is also assumed that a damping ratio of the system equals *α* = 10% [14]. For comparison purposes, three types of soil, (i) Pasternak soil type, (ii) Pasternak-Vlasov soil type, and (iii) Pasternak-Vlasov accounting the soil inertia type (three-parameter soil type), are considered.

### 3.1. Variation of Deflection as a Function of Time

**Figure 2:**
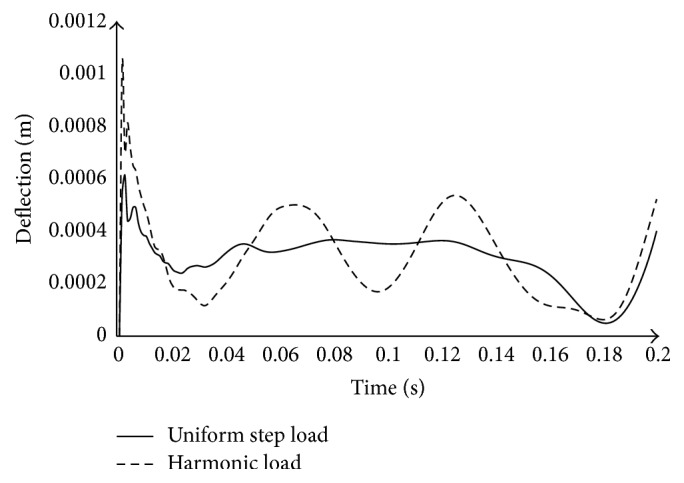
Variation of the deflection directly under load versus time for different types of load (*H*_*s*_ = 2.5 m).

Figure 2 shows, for the three-parameter soil, the variation of deflection under load as a function of time. This figure shows the time-displacement curve for two types of load: the uniform step load and the harmonically load. These loads evolve with the same constant acceleration and initial velocity. It is found that the deflection of the plate initially greatly increases with rapid oscillations and high amplitude, up to the moment *t* = 3.5 × 10^−2^ s. This observation characterizes the transient domain for both types of load. After this phase, a stabilization of the oscillations is noted and the plate enters the stationary domain. In the stationary domain, the amplitude of deflection does not vary depending on the time. For harmonic load, the deflection varies harmonically with the time, since this load type is harmonic; but for step load, the deflection does not vary considerably in stationary domain. Yet, the deflection seemed affected by the boundary condition, since the deflection varies considerably for both types of load when the load approaches boundary of the plate. The maximum of deflection in transient domain appears at time *t* = 0.003 s and this value is 66 percent more than those in stationary domain which appears at time *t* = 4*π*/*ω* = 0.1256 s for harmonic load. This shows how it is important to take into account the transient domain response in the rigid pavement structures resistance analysis. In the specific case, the part of the plate concerned by this deflection is the segment [0; 0.87] m. Beyond these points, the value of maximum deflection is *w* = 0.000551479 m. Besides, it is noticed that the response of uniform concentrated time step load is less than the harmonic load. That occurs in both transient domain and steady-state domain of plate response.

**Figure 3:**
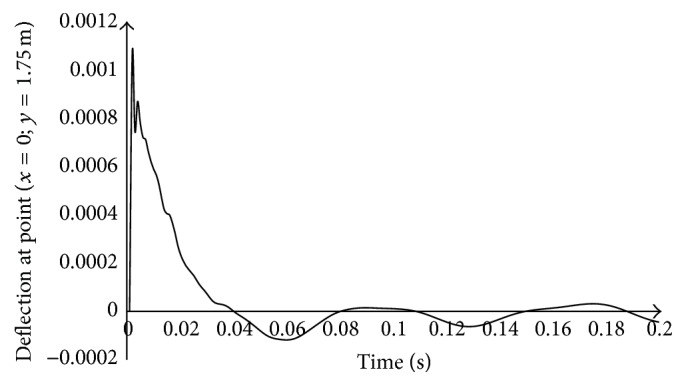
Variation of the deflection at the fixed point of coordinates (*x* = 0; *y* = 1.75 m), versus time for a dynamically activated soil depth: *H*_*s*_ = 2.5 m.

Figure 3 shows the variation of the deflection as a function of time at a fixed point with coordinates *x* = 0 and *y* = 1.75 m, when the load is moving along the central axis of the plate (0 ≤ *x* ≤ 5 m and *y* = 1.75 m). It is noted that the maximum deflection obtained neighborhood observation point along the traveling direction of the load and decreases in magnitude as well, as the load is removing. This shows that the maximum deflection occurs near the application point of the load.

### 3.2. Dynamically Activated Soil Depth (*H*_*s*_) Effect on Dynamic Response

Based on the data listed above, the first five mode numbers of the plate modeling the pavement were determined in the *x*-direction and the first five mode numbers were determined in the *y*-direction. We plotted the variation of the deflection curves according to different variables: *x*, *y*, and *t*.

**Figure 4:**
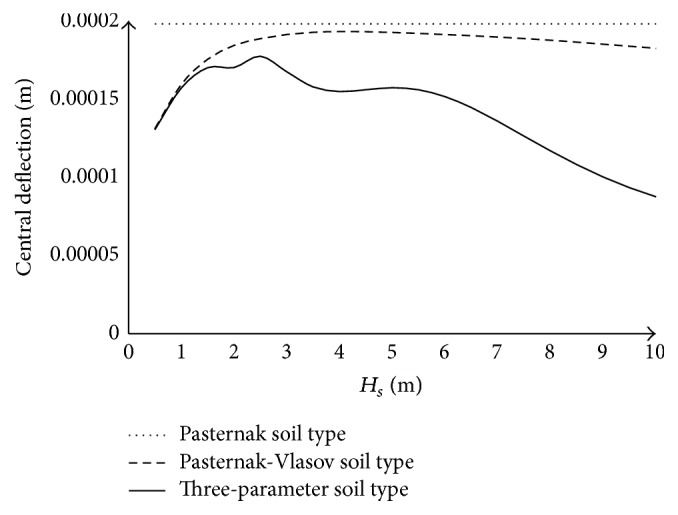
Variation of the deflection in the center of the plate (*x* = 2.5 m, *y* = 1.75 m) as a function of the dynamically activated depth of soil at time *t* = 0.099603 s when the load is located at the center of the plate.

Figure 4 shows the variations of the deflection at the center of the plate (*x* = 2.5 m; *y* = 1.75 m) depending on the dynamically activated soil depth for different types of soil when the load is located at the center of the plate. It can be seen that, for the Pasternak soil, the value of the deflection of the plate is constant (*w* = 0.000197106 m) whatever the considered depth of the foundation. For Pasternak-Vlasov soil type and three-parameter soil type, the deflection increases to reach a maximum value at a given depth (*H*_*s*_ = 4.0 m for Vlasov soil type with *w* = 0.000192939 m and *H*_*s*_ = 2.5 m for three-parameter soil type with *w* = 0.000157565 m). For lower values of *H*_*s*_ up to *H*_*s*_ = 1.25 m, the deflection of the two types of soil is the same. After this depth, the value of the deflection for three-parameter soil type is less than those of Vlasov soil type. The difference between the two responses increases with the depth of the dynamically activated soil up to 52 percent at *H*_*s*_ = 10 m.

It is deduced from these observations that the dynamically activated soil depth greatly influences the response of the plate. We have chosen in this study the depth maximizing the three-parameter soil type; *H*_*s*_ = 2.5 m.

### 3.3. Influence of the Inertia of the Soil on the Dynamic Response

**Figure 5:**
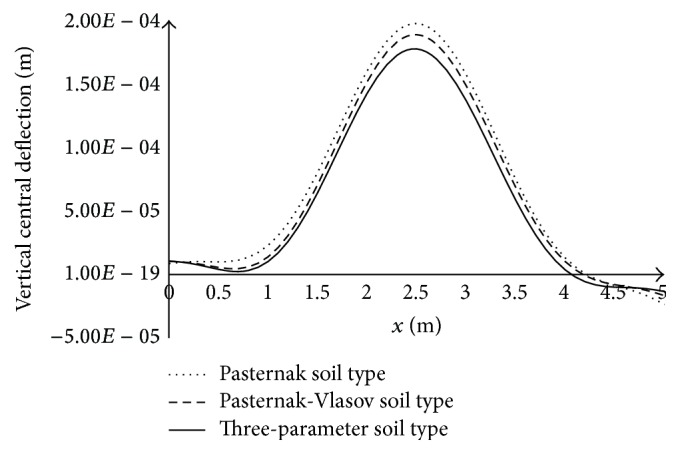
Variation of the deflection along the central axis of the plate (0 ≤ *x* ≤ 5 m; *y* = 1.75 m), for different types of soil, at time *t* = 0.099603 s, when the load is located at the center of the plate.

Figure 5 shows the changes in the deflection along the central axis of the pavement plate (*y* = 1.75 m; 0 ≤ *x* ≤ 5 m), for different types of soil, at time *t* = 0.099603 s when the moving load arrived at the center of the plate (*x* = 2.5 m and *y* = 1.75 m). It is noticed that the deflection is larger throughout the plate considering the Pasternak soil, compared to the deflection values for the three-parameter soil. The deflections of the plate when the soil is Pasternak-Vlasov type have values between those obtained for the three-parameter soil and Pasternak soil types. Taking the values of the deflections of the plate on the Pasternak-Vlasov soil type as a reference, it can be seen that the deflections at the center of the plate are reduced up to 18.33 percent. The more the depth *H*_*s*_ is, the higher the gap is. In conclusion, inertial soil greatly reduces (up to 18.33%) the dynamic response of the pavement plate when the moving load is over the center of the plate.

### 3.4. Effect of the Variation of Load Magnitude on the Displacement for Different Values of Dynamically Activated Depth

**Figure 6:**
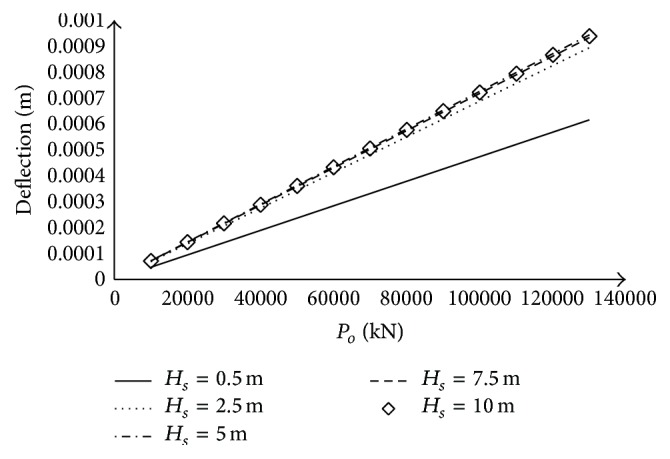
Variation of the deflection as a function of plate magnitude, for different values of dynamically activated soil depth, at time *t* = 0.1256 s.

Figure 6 shows the deflection of the plate versus the load magnitude for dynamically activated soil depths: *H*_*s*_ = 0.5 m; 2.5 m; 5 m; 7.5 m; 10 m. It is noticed that the displacement increases linearly with the increase in load magnitude. Besides, the increasing of the dynamically activated depth parameter *H*_*s*_ increases the deflection of the plate for low values of *H*_*s*_. Nevertheless, for great values of dynamically activated load, the deflection of the plate is not affected by the variation of the dynamically activated depth.

### 3.5. Variation of Displacement at Optimal Time versus Soil Parameters for Different Values of *b*/*a*

Table 1 presents the values of displacement under load versus soil parameters for different values of *b*/*a* (*b*/*a* = 0.5; *b*/*a* = 0.7; *b*/*a* = 1.0) at time *t* = 0.1256 s. This table is obtained for plates covering the same area. The table shows that deflection increases with the increase in *b*/*a*. So the deflection is great for square plate compared to the rectangular plate. The size of pavement plate considerably affected the displacement of the pavement plate. It is more useful for engineer to design the rigid pavement plate as a rectangular one.

## 4. Conclusion

This paper dealt with some significant results from a study of the dynamic analysis of rigid pavements. The soil models used in this work are the well-known Pasternak model, the Pasternak-Vlasov model which takes into account the interaction between soil layers, and the improved three-parameter model considering the inertia of the soil. The main conclusions of this study are the following:

(i) The soil inertia influences the pavement response at the middle of the plate when the load evolving along its centerline arrived at the center. This indicates a possible overdesign of pavements when using the two-parameter soil model.
(ii) The effect of dynamically activated depth of Pasternak-Vlasov soil and three-parameter soil on the response is found to be significant for both soils types but more for three-parameter type than the Pasternak-Vlasov type.
(iii) Before the stationary domain of oscillations, a transient response of the plate during 3.5 × 10^−2^ seconds is noted. The transient response is bigger than the stationary one and it will be necessary to investigate deeply in further study.
(iv) This study only covers plates pavement interconnected by dowels and tie bars. So, we could extend it to continuous pavements plates.
(v) This study does not take into account the cyclic effect of the load. So, the fatigue response of the studied system could be further analyzed.
(vi) The resonance is not studied in this work despite the effect that soil inertia can have in that, so we intend to study it in further work.

## Figures and Tables

**Table 1:** Displacement versus soils parameters for different values of *b*/*a* (*b*/*a* = 0.5; *b*/*a* = 0.7; *b*/*a* = 1.0) at time *t* = 0.1256 s.

*b*/*a*	*b*/*a* = 0.5	*b*/*a* = 0.7	*b*/*a* = 1.0
*H*_*s*_ = 0.5			
*k*_*o*_ = 1.662 × 10^8^	3.77 × 10^−4^ m	3.78 × 10^−4^ m	5.73 × 10^−4^ m
*C*_*o*_ = 2.596 × 10^6^			
*m*_*o*_ = 252.326			

*H*_*s*_ = 2.5			
*k*_*o*_ = 3.325 × 10^7^	5.27 × 10^−4^ m	5.50 × 10^−4^ m	7.08 × 10^−4^ m
*C*_*o*_ = 1.298 × 10^7^			
*m*_*o*_ = 1261.63			

*H*_*s*_ = 5			
*k*_*o*_ = 1.662 × 10^7^	5.36 × 10^−4^ m	5.8 × 10^−4^ m	7.81 × 10^−4^ m
*C*_*o*_ = 2.596 × 10^7^			
*m*_*o*_ = 2523.26			

*H*_*s*_ = 7.5			
*k*_*o*_ = 1.108 × 10^7^	5.48 × 10^−4^ m	5.73 × 10^−4^ m	8.48 × 10^−4^ m
*C*_*o*_ = 3.894 × 10^7^			
*m*_*o*_ = 3784.89			

*H*_*s*_ = 10			
*k*_*o*_ = 8.311 × 10^6^	5.13 × 10^−4^ m	5.77 × 10^−4^ m	9.37 × 10^−4^ m
*C*_*o*_ = 5.192 × 10^7^			
*m*_*o*_ = 5046.52			

## References

[1] Rahman S. O., Anam I. (2005). Dynamic analysis of concrete pavement under moving loads. *Journal of Civil and Environmental Engineering*.

[2] Wei Tu M. S. Response modelling of pavement subjected to dynamic surface loading based on stress-based multi-layered plate theory.

[3] LCPC (1994). *Conception et Dimensionnement des Chaussées Neuves: Guide Technique*.

[4] Baus R. L., Stires N. R., Carolina U. O. S. (2010). *Mechanistic-Empirical Pavement Design Guide*.

[5] Huang M.-H., Thambiratnam D. P. (2001). Deflection response of plate on Winkler foundation to moving accelerated loads. *Engineering Structures*.

[6] Kirn S.-M., Roesset J. M. (1998). Moving loads on a plate on elastic foundation. *Journal of Engineering Mechanics*.

[7] Ullidtz P. (1998). *Modelling Flexible Pavement Response and Performance*.

[8] Sun L. (2007). Steady-state dynamic response of a Kirchhoff's slab on viscoelastic Kelvin's foundation to moving harmonic loads. *Journal of Applied Mechanics*.

[9] St-Laurent D. (2008). *Synthèse des Outils de Modélisation de Chausssées Actuellement Disponibles au LCPC*.

[10] Lu S. (2006). Analytical dynamic displacement response of rigid pavements to moving concentrated and line loads. *International Journal of Solids and Structures*.

[11] ARA (2003). Appendix QQ: structural response models for rigid pavements. *Guide for Mechanistic-Empirical Design of New and Rehabilitated Pavement Structures*.

[12] Nguyen-Thoi T., Luong-Van H., Phung-Van P., Rabczuk T., Tran-Trung D. (2013). Dynamic responses of composite plates on the pasternak foundation subjected to a moving mass by a cell-based smoothed discrete shear gap (CS-FEM-DSG3) method. *International Journal of Composite Materials*.

[13] Aron C., Jonas E. (2012). *Structural element approaches for soil-structure interaction [M.S. thesis]*.

[14] Alisjahbana S., Wangsadinata W. (2012). Dynamic analysis of rigid roadway pavement under moving traffic loads with variable velocity. *Interaction and Multiscale Mechanics*.

[15] Lu S. (2001). Dynamic displacement response of beam-type structures to moving line loads. *International Journal of Solids and Structures*.

[16] Civalek Ö. (2007). Nonlinear analysis of thin rectangular plates on Winkler–Pasternak elastic foundations by DSC–HDQ methods. *Applied Mathematical Modelling*.

[17] Pan G., Atluri S. N. (1995). Dynamic response of finite sized elastic runways subjected to moving loads: a coupled BEM/FEM approach. *International Journal for Numerical Methods in Engineering*.

[18] Gibigaye M. (1992). *Problems of Dynamic Response of Shells Contacting with an Inertial Medium, at a Transient Domain of Vibrations*.

[19] Dimitrovová Z. Enhanced formula for a critical velocity of a uniformly moving load.

[20] Asik M. S. Vertical vibration analysis of rigid footings on a soil layer with a rigid base. https://repositories.tdl.org/ttu-ir/bitstream/handle/2346/20107/31295007715294.pdf?sequence.

[21] Turhan A. (1992). *A Consistent Vlasov Model for Analysis of Plates on Elastic Foundations Using the Finite Element Method*.

[22] Xiang-sheng C. (1987). Dynamic response of plates on elastic foundations due to the moving loads. *Applied Mathematics and Mechanics*.

[23] Samul V. I. (1982). *The Basis of Elasticity and Plasticity Theories: Pedagogic Book for High Schools Students*.

[24] Harberman R. (1987). *Elementary Applied Partial Differential Equations with Fourier Serie and Boundary Value Problem*.

[25] Alisjahbana S. Dynamic response of clamped orthotropic plates to dynamic moving loads.

